# Enhancing human phenotype ontology term extraction through synthetic case reports and embedding-based retrieval: A novel approach for improved biomedical data annotation

**DOI:** 10.1016/j.jpi.2024.100409

**Published:** 2024-11-16

**Authors:** Abdulkadir Albayrak, Yao Xiao, Piyush Mukherjee, Sarah S. Barnett, Cherisse A. Marcou, Steven N. Hart

**Affiliations:** a Department of Laboratory Medicine and Pathology, Mayo Clinic, Rochester, MN, United States of America; b Department of Quantitative Health Sciences, Mayo Clinic, Rochester, MN, United States of America; c Center for Digital Health, Mayo Clinic, Rochester, MN, United States of America

**Keywords:** Human phenotype ontology, PhenoTagger, Vector embeddings

## Abstract

With the increasing utilization of exome and genome sequencing in clinical and research genetics, accurate and automated extraction of human phenotype ontology (HPO) terms from clinical texts has become imperative. Traditional methods for HPO term extraction, such as PhenoTagger, often face limitations in coverage and precision. In this study, we propose a novel approach that leverages large language models (LLMs) to generate synthetic sentences with clinical context, which were semantically encoded into vector embeddings. These embeddings are linked to HPO terms, creating a robust knowledgebase that facilitates precise information retrieval. Our method circumvents the known issue of LLM hallucinations by storing and querying these embeddings within a true database, ensuring accurate context matching without the need for a predictive model. We evaluated the performance of three different embedding models, all of which demonstrated substantial improvements over PhenoTagger. Top recall (sensitivity), precision (positive-predictive value, PPV), and F1 are 0.64, 0.64, and 0.64, respectively, which were 31%, 10%, and 21% better than PhenoTagger. Furthermore, optimal performance was achieved when we combined the best performing embedding model with PhenoTagger (a.k.a. Fused model), resulting in recall (sensitivity), precision (PPV), and F1 values of 0.7, 0.7, and 0.7, respectively, which are 10%, 10%, and 10% better than the best embedding models. Our findings underscore the potential of this integrated approach to enhance the precision and reliability of HPO term extraction, offering a scalable and effective solution for biomedical data annotation.

## Introduction

Healthcare is a data-driven practice, where streamlined access to well-curated information is a prerequisite to decision making. Advances in sequencing technologies and bioinformatic workflows have dramatically increased the use of genomic data in clinical diagnostics and translational research, driving the practice of genomically informed medicine. The clinical utility of comprehensive, genome-wide exploratory exome and genome sequencing is well documented and is increasingly becoming a first-line diagnostic strategy for individuals with suspected genetic disease.^1^ Additionally, the increasing affordability and scalability of genomic testing has enabled widespread applications across populations and healthcare domains,^2^ advancing the promise of personalized medicine.

Exome and genome sequencing generate vast amounts of data, needing automated and performant bioinformatics resources for interpretation. Analyzing large numbers of variants in a patient's genome poses challenges due to the volume and complexity of the genetic data and the necessity of well-defined phenotypic information to guide interpretation. The integration of automated and integrated solutions to extract structured patient phenotypes from what is commonly unstructured free text is often limited in current genomic workflow solutions. The human phenotype ontology (HPO)^3^^,^^4^ is the primary resource used in clinical genetics and diagnostics to describe structured phenotypes. Represented as HPO terms, these structured phenotypes are a critical input into workflows for genomic data interpretation, whereby curated gene–phenotype associations are leveraged to prioritize genomic variants for interpretation.^5^ As such, extracting HPO terms from clinical texts is essential because such terms provide a standardized vocabulary that enables the precise characterization of patient phenotypes, which in turn supports effective diagnosis, treatment, and research. The systematic extraction of HPO terms from unstructured clinical texts ensures that phenotypic information is captured efficiently, which is essential for linking genetic variants to clinical presentations.

Current practices of phenotypic term extraction in the absence of automation are labor-intensive and put a significant burden on subject matter experts in the diagnostic lab. This is inherently at odds with the increasing utilization of genomic sequencing across many healthcare specialties which requires scalability of solutions to meet market demands. Consequently, there exists a critical need to find solutions to automate the phenotypic extraction process, thereby meeting the needs of the diagnostic lab, enhancing the scalability and accuracy of genetic diagnoses, and enabling better patient outcomes.

There are multiple tools that have improved computational extraction and subsequent utilization of structured phenotypic data encoded as HPO terms. Examples include PhenoTagger and PhenoBERT, which leverage advanced natural language processing (NLP) techniques to extract and encode phenotype information from textual data, enhancing accuracy and efficiency.^6^^,^^7^ Tools like MetaMap and NCBO offer robust semantic annotation capabilities, facilitating interoperability and standardization across different datasets and studies.^8^^,^^9^ OBO and Doc2Hpo streamline ontology-based annotation and mapping, ensuring consistent phenotype descriptions.^10^ ClinPhen and NCR focus on clinical data integration, supporting comprehensive phenotype characterization.^11^ Finally, BERN210 and JSL11 contribute to machine learning-driven phenotype prioritization, aiding in the identification of clinically relevant phenotypes with high precision. These tools all use advanced computational techniques to enhance accuracy, efficiency, and interoperability in the identification and prioritization of HPO terms in areas of clinical diagnostic testing and research.

Several limitations exist in the tools used for HPO term identification from unstructured text including a lack of sensitivity in identifying newer or less common HPO terms.^12^ A decreased accuracy in HPO term identification is observed when discerning between similar but contextually distinct terms, yielding incorrect term associations.^4^ Also observed are challenges with NLP methods correctly assigning HPO terms to an individual when they are complicated by concepts like familial relationships or negation. In addition, integration of these tools into existing biomedical workflows or databases can be challenging due to differences in data formats, APIs, or computational requirements. Together, these limitations lead to issues of incomplete phenotypic extraction, poor generalization across document sets, and an overall lower accuracy in HPO term identification in a clinical setting.

Leveraging advanced artificial intelligence techniques has emerged as a promising strategy to address some of these challenges in HPO term extraction. One such technique is retrieval-augmented generation (RAG), which stands out for its ability to semantically encode information in vector databases.^13^ This approach uses embeddings to represent textual data in a high-dimensional space, allowing for sophisticated semantic analysis. Unlike traditional root–stem-based methods, which primarily focus on morphological patterns and syntactic structures,^14^ RAG employs cosine similarity measures to identify and extract semantically meaningful relationships between terms. This fundamental shift in methodology opens new possibilities for improving HPO extraction by enhancing the contextual understanding of phenotype descriptions.

In this study, we evaluated whether RAG semantic similarity search can accurately identify HPO terms from unstructured text. By utilizing RAG's vector-based semantic encoding and similarity measures, it is hypothesized that we can achieve a significant improvement in finding and relating HPO terms in diverse textual contexts. We tested whether this approach would complement traditional extraction methods, overcoming some of the known limitations and resulting in an integrated solution with more performant F1 scores compared to traditional methods alone. The integration of RAG techniques into HPO extraction workflows is a promising advancement, poised to enhance the precision and efficacy of phenotype data analysis in various research and clinical applications.

## Methods

Traditional NLP-based HPO term extraction tools rely on specific online databases and curated datasets, targeting phenotype data from resources to perform defined tasks with structured annotations. To provide more comprehensive solutions to understand and capture phenotypic information from textual data, large language models (LLMs) are trained on diverse datasets, including the HPO dictionary, enabling them to capture nuanced relationships and meanings within phenotypic concepts. This broad training gives LLMs a more holistic understanding of HPO terms, making them valuable for precise phenotypic information generation in clinical contexts. We proposed a new method that includes generation of clinical free text using ChatGPT 3.5, using embedding models to encode the phenotypic information and fusing the state of the art NLP-based approach with an LLM-based approach to overcome these limitations.

### LLM-based method for HPO term extraction

#### Generating N sentences for each HPO term

**Fig. 1:**
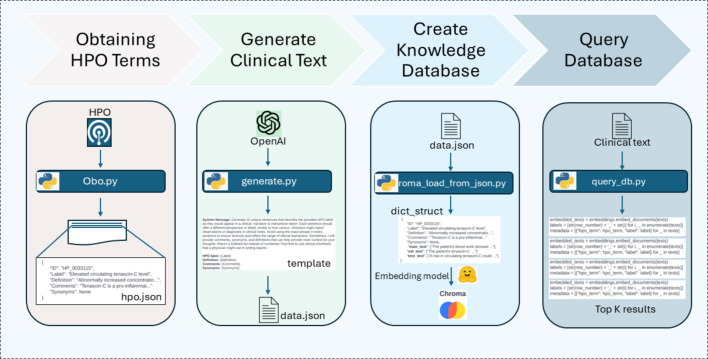
Prediction of HPO terms from clinical text workflow includes the following stages: obtaining HPO terms, generating clinical text, creating the knowledge database, and querying the database.

The first step in building a RAG-based method for HPO term extraction is to extract the 18,696 HPO terms from the official HPO website^15^ (Fig. 1). Using the HPO code, HPO label, definition, synonyms, and comments data, we generated 40 sentences where that term was used in a clinical context using ChatGPT 3.5. A prompt template was constructed to provide the proper context for each term and appeared as follows:

“*Generate 40 unique sentences that describe the provided HPO label as they would appear in a clinical narrative or interpretive report. Each sentence should offer a different perspective or detail, similar to how various clinicians might report observations or diagnoses in clinical notes. Avoid using the exact phrase in every sentence to ensure diversity and reflect the range of clinical expression. Sometimes, I will provide comments, synonyms, and definitions that can help provide more context for your thoughts. Return a bulleted list instead of numbered. Feel free to use clinical shorthand that a physician might use in writing reports.*

*HPO label: <Label>*

*Definition: <Definition>*

*Comments: <Comments>*

*Synonyms: <Synonyms>*”

Entries enclosed in angle brackets represent variables or placeholders intended to be replaced with specific values or data depending on the context. Forty sentences were constructed for each HPO term. In total, 458,574 sentences were generated for 18,696 HPO terms using ChatGPT 3.5.

#### Creating knowledge database

The first 32 sentences per term were transformed into embeddings for insertion into a ChromaDB database,^16^ using cosine similarity as the distance function. ChromaDB is an open-source database that is specifically designed for storing and using vector embeddings with their relevant metadata. Specifically, we inserted the source HPO materials into the metadata fields which were used downstream. Despite requesting 40 sentences, the LLM often generated fewer. This is not surprising as this generation of LLMs are well known to underperform at basic counting function without the aid of external tooling. To ensure consistent representation for each HPO term, only the first 34 sentences were used (32 to build the knowledgebase and 2 for testing). Three embedding models were tested: HKUNLP (hkunlp/instructor-large),^17^ MPNET-Base-V2,^18^ and multiQA-MiniLM-cos-v1,^19^ as their model weights are available to be downloaded and run locally, without an external API call. We refer to this ChromaDB as the reference “knowledge base”. The remaining two generated sentences per HPO term (*n* = 37,392), were used as a hold-out test set.

#### Similarity measurement by querying the knowledge database

To query the system, each of these sentences was embedded, searched against the nearest synthetic sentence in the knowledge base via cosine similarity, and extracting the HPO term from the metadata for the top *k* closest matches. We define this process as the Embedding Model (EM). Performance metrics such as sensitivity, positive-predictive value (PPV), and F1-score were calculated on the 27,392 hold-out test set to assess how well the system retrieves relevant information. The F1 score is the harmonic mean of precision and recall, balancing the trade-off between these two metrics. This is particularly valuable in biomedical data annotation, where both false positives (FP) and false negatives (FN) can have significant downstream consequences.

### PhenoTagger NLP-based HPO term extraction

PhenoTagger (PT, v1.2) is a hybrid method that combines both dictionary and machine learning-based methods to recognize HPO concepts in unstructured biomedical text. Input text was processed through NLP pipelines, which include tokenization, part-of-speech tagging, and named entity recognition to identify and extract relevant phenotypic information. It then applied a dictionary-based method to map phenotypic information to HPO concepts. Each sentence input was predicted directly. Default parameters settings were used for all experiments.

### Improving performance by building a Fused Model

To enhance classification accuracy, a decision fusion^20^ approach was developed that integrates predictions from PT and EM models. Each model independently generates classification results based on the generated given input sentence. The final classification outcome is determined by combining the top *k* results from both models. Here, *k* is chosen as 1 and 5 for the most similar sentences' label. This combined approach leverages the strengths of both models, aiming to improve overall classification reliability and robustness. This approach is referred to as the Fused Model (FM).

As an example, Table 1 represents the top five results that were obtained from the PT and EM models. The PT model yielded predictions consisting of four instances of “HP:0000002” and one instance of “HP:0000098”. Conversely, the EM produced predictions of two instances of “HP:0000002” and three instances of “HP:0000098”. The FM aggregates the predicted labels from each model to determine the final classification via a majority vote across the predictions from both models, resulting in the final classification being the label with the highest combined count. This approach ensures that the strengths of each model are leveraged, aiming to improve the robustness and accuracy of the final classification outcome. In the case of *k* = 1, that HPO id is directly assigned as the result. In case of different results, the result of the PT model is accepted as correct.

**Table 1:** An example clinical note obtained from our generated dataset. The responses include Top five HPO IDs obtained from Embedding model (MPNET-Base-V2) and PT model. The final decision is made according to majority voting across the predictions from both models. Items in red represents the true HPO term.

“001|t|Patient idx: 001001|a|notes: *Clinically significant discrepancy in height observed between patient and their peers.*” (Ground Truth: HP:0000002)	
Model	Response
MPNET-Base-V2 Response	**HP:0000002, HP:0000002, HP:0000098, HP:0000002, HP:0000002,**
PT Model Response	**HP:0000002, HP:0000098, HP:0000002, HP:0000098, HP:0000098**
FM	**HP:0000002**

### Evaluation metric definitions

True positives (TP) represent the number of correctly identified sentences where the predicted HPO Term ID matches the actual one. FP are sentences incorrectly classified by the model as having another specific HPO Term ID. FN were incorrectly classified as not having a certain HPO Term ID when they do. Sensitivity, PPV, and F1 measures are derived from these definitions that collectively offer a comprehensive evaluation of our classification system's performance in accurately assigning HPO Term IDs to sentences.

## Results

### Evaluation of different embedding models

**Fig. 2:**
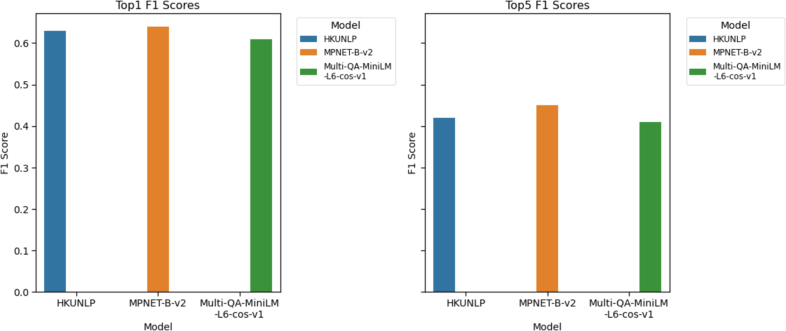
F1 scores comparison of the different embedding models depends on Top 1 and Top 5 scores.

HKUNLP represents a substantial embedding model that underwent training on an extensive corpus of text, boasting a model weight of approximately 4.6 gigabytes. The model requires a quite large amount of physical space while comparing alternative ones. MPNET and multiQA-MiniLM-cos-v1 relatively less space at 0.41 and 0.09 GB, respectively. Although smaller, they exhibit similar performances as HKUNLP (Fig. 2**,** F1-score: 0.64), therefore MPNET was used as the embedding of choice.

### Evaluation of models

The embedding strategy, with the MPNET embeddings, was then compared to the more traditional NLP-based method PT. Fig. 2 illustrates the F1 score performance metrics which vary based on the top N retrieved HPO terms. Top 1 denotes the single HPO Term to be retrieved for every input from the knowledge database, whereas Top 5 denotes the five HPO Terms in the nearest neighbors obtained from the database for the provided input. When the results obtained are evaluated with respect to Top 1, MPNET-Base-V2 shows superior performance when compared to the other models, particularly in terms of the F1 score. Although the PT model gives similar results in sensitivity, it performs poorly in PPV (Table 2).

**Table 2:** Model performance of PT and FM on the test set.

Model name	TopK	TP	FP	FN	Sensitivity	PPV	F1
PhenoTagger	Top1	18,253	13,258	19,139	0.49	0.58	0.53
PhenoTagger	Top5	22,984	40,533	14,408	0.62	0.36	0.46
FM	Top1 (only)	25,170	10,683	10,532	0.70	0.70	**0.70**

To further improve the results, an approach was devised to combine the decisions of the PT model and the EM. This FM, defined in the methods, yielded the highest F1 score overall at 0.70. To explain why the FM demonstrated improvement over PT, we can refer to the example in Table 1. PT assumed a majority prediction for HP:0000098, but also correctly called the ground truth HP:0000002. Using the logic of the FM, only one majority could be selected – in this case the actual HPO term HP:0000002.

The experiments were performed using a computer equipped with an 11th Gen Intel® Core™ i5-1145G7 processor (64-bit), operating at a base clock speed of 2.60 GHz, paired with 16 GB of RAM. The total time required for creating each embedding model's knowledge database was as follows: 2337 min for HKUNLP, 582 for MPNET minutes, and 141 min for multiQA-MiniLM-cos-v1.

## Discussion

Our primary contribution is the creation of a new modality for HPO term extraction. We have shown how LLMs can be used to generate useful input data rather than painstaking curation by experts to manually link sentences to HPO terms. At the scale of the entire HPO database, this would be impractical without multinational collaboration and funding. Second, by encoding a linkage between the embedding vectors and HPO terms, we create a semantic bridge that can be exploited to leverage the context similarity provided by embedding models and the metadata-based tagging within databases for accurate information retrieval. This contrasts with other approaches that may try to exclusively use LLMs as databases. The issue of hallucinations is a well-known artifact of LLMs,^21^, ^22^, ^23^ and therefore operators must use discretion when they are or are not appropriate to use. In this approach, the role of the LLM is solely to hallucinate imaginary case reports for each HPO term.

To leverage the information from the hallucinated knowledge base, the embeddings and their HPO metadata are housed in a true database. Importantly, there is no training nor a predictive model necessary for downstream use. By querying the embedding of a test question, the database finds the nearest similar context and returns the HPO tag associated with it. This prevents an LLM from hallucinating an incorrect HPO term, while simultaneously identifying relevant contexts based on semantic meaning instead of relying on regular expressions or other NLP-based methods.

We demonstrated the effectiveness of this strategy by comparing different embedding models and benchmarking against PT. Only minor differences were observed with different embedding models, but a dramatic improvement was also demonstrated over PT. However, we also noticed that the combination of the strengths of PT with the strengths of the embedding strategy had complementary effects. Thus, by combining these two approaches into the FM, we demonstrate improved performance than either tool alone.

One limitation of this approach is that it relies on full-text sentences as input. However, sometimes only keywords might be provided, in which case cosine similarity might not be the best choice, in such cases, it would be interesting to try different distance metrics like L2 or inner product may be a better option. More studies are required to vet this hypothesis. Also, we note that the data presented here are generated entirely from LLMs and, despite our best efforts, may not reflect real-world data. We believe this limitation to be addressable though, if real-world data were to be summarized by the same LLM used when generating synthetic summaries. Privacy risks aside, this would at least make the data conform to the same statistical properties as the embeddings and therefore should be directly comparable.

## Conclusion

In conclusion, our study introduces a novel approach for HPO term extraction by leveraging LLMs to generate synthetic sentences with clinical context that can be utilized as a knowledgebase, thereby reducing the need for extensive manual curation. By embedding a linkage between vector representations and HPO terms, we create a semantic bridge that facilitates accurate information retrieval, whereas mitigating the risk of LLM-induced hallucinations. This method not only enhances the efficiency of HPO term extraction but also outperforms traditional NLP-based approaches, like PhenoTagger. However, when combined together in a FM, the precision and accuracy of HPO term extraction is even higher. Our findings highlight the potential of this integrated approach to significantly improve the precision and reliability of HPO term extraction, paving the way for more scalable and effective biomedical data annotation techniques.

## Declaration of competing interest

The authors declare that they have no known competing financial interests or personal relationships that could have appeared to influence the work reported in this article.
